# Design of (Nb, Mo)_40_Ti_30_Ni_30_ alloy membranes for combined enhancement of hydrogen permeability and embrittlement resistance

**DOI:** 10.1038/s41598-017-00335-0

**Published:** 2017-03-16

**Authors:** Xinzhong Li, Xiao Liang, Dongmei Liu, Ruirun Chen, Feifei Huang, Rui Wang, Markus Rettenmayr, Yanqing Su, Jingjie Guo, Hengzhi Fu

**Affiliations:** 1 School of Materials Science and Engineering, Harbin Institute of Technology, Harbin, 150001 PR China; 2 Otto Schott Institute of Materials Research, Friedrich Schiller University, Jena, 07743 Germany

## Abstract

The effect of substitution of Nb by Mo in Nb_40_Ti_30_Ni_30_ was investigated with respect to microstructural features and hydrogen dissolution, diffusion and permeation. As-cast Nb_40−x_Mo_x_Ti_30_Ni_30_ (x = 0, 5, 10) alloys consist of primary bcc-Nb phase and binary eutectic (bcc-Nb + B2-TiNi). The substitution of Nb by Mo reduces the hydrogen solubility in alloys, but may increase (x = 5) or decrease (x = 10) the apparent hydrogen diffusivity and permeability. As-cast Nb_35_Mo_5_Ti_30_Ni_30_ exhibits a combined enhancement of hydrogen permeability and embrittlement resistance as compared to Nb_40_Ti_30_Ni_30_. This work confirms that Mo is a desirable alloying element in Nb that can contribute to a reduction in hydrogen absorption and an increase in intrinsic hydrogen diffusion, thus improving embrittlement resistance with minimal permeability penalty.

## Introduction

The mass production of high purity hydrogen is one of the central topics of hydrogen economy, which has attracted much attention in recent years. Membrane separation is the benchmark technology used to separate and purify hydrogen from a mixed or contaminated gas, typically produced by methane steam reforming^1–5^ or coal gasification^6–8^. Foremost among the possible choices of metallic membrane materials are Pd-based alloys due to their high catalytic surface property, reasonable hydrogen permeability, good durability and tolerance to syngas species. It was reported in refs 9 and 10 that a nominal hydrogen production capacity of 40 Nm^3^/h and an energy efficiency of 70–76% could be reached by a modern steam reforming system with Pd-based alloy membranes. Recently, Pd-based alloy membranes were extended to produce ultra-pure hydrogen by catalytic reforming of olive mill wastewater (OMW)^11, 12^. OMW is a biomass by-product of the olive oil industry, which results in environmental issues because of its poor biodegradability and high phytotoxicity. A treatment of OMW in a Pd-Ag membrane reactor demonstrated the capability to produce up to 3.25 kg of ultra-pure hydrogen per ton of OMW^11^. A higher capability in hydrogen production of 12.3 kg per ton of OMW was reached by catalytic reforming of the combined OMW and methane in a Pd-Ag membrane reactor^12^. However, the cost of Pd is uneconomically high and subject to dramatic changes, which restricts its large scale application. This stimulates the development of pinhole-free Pd-based alloy membranes with ever decreasing thickness^13^, or the development of new less expensive metal membranes.

Because of higher hydrogen permeability but much lower cost and richer natural resources, group V metals, such as vanadium (V), niobium (Nb) and tantalum (Ta) are among the leading candidates to replace Pd-based alloys for hydrogen separation. In pure form, they are sensitive to hydrogen embrittlement (HE) induced by excessive hydrogen absorption. Much of current research is focused on compositional or/and structural modification to inhibit HE. This generates mainly two types of notable hydrogen permeable alloys. The first type comprises V^14–20^, Nb^21–24^ and Ta^25^ solid solution alloys formed by selective alloying for group V metals with the aim to reduce hydrogen solubility. Such alloys exhibit an obviously improved HE resistance as compared to the pure counterpart, and also show distinctly higher hydrogen permeability than the leading Pd-based alloys. The other type comprises dual-phase alloys such as Nb-Ti-Ni^26–31^, Nb-Ti-Co^32–36^, V-Ti-Ni^37–39^, Ta-Ti-Ni^40, 41^ etc. These alloys generally consist of primary bcc-Nb/V/Ta for hydrogen permeation and secondary eutectic (bcc-Nb/V/Ta + B2-TiNi/TiCo) with high HE resistance. After pertinent modulation of the dual-phase structure, these alloys achieve an excellent balance between hydrogen permeability and embrittlement resistance.

Overall, there still remain several challenges for the above mentioned V, Nb and Ta based alloys to meet the US DOE performance targets^42, 43^ for hydrogen separation and purification applications. Meeting hydrogen permeation flux and durability targets are the key issues to be solved for these materials. One strategy is compositional modification for tailoring hydrogen solubility and diffusivity. The desirable effect is a reduction in hydrogen solubility and an increase in hydrogen diffusivity, thus improving the HE resistance with no or minimal permeability penalty. However, it was found that the hydrogen diffusion through V-Ni^44^, V-Ni-Ti^45^, Nb-Ti-Co^36^ etc. exhibits a significantly concentration dependent behavior. Decreasing hydrogen solubility is generally coupled with a reduced hydrogen diffusivity. This is in contradiction with the above mentioned aim of compositional modification.

Recently, Yukawa *et al.* ^46, 47^ reported that the addition of W and Mo in Nb could reduce hydrogen solubility and simultaneously increase hydrogen diffusivity. They presented an Nb-8W-8Mo alloy membrane that shows significantly enhanced HE resistance and hydrogen flux, which is, however, only applicable in a limited hydrogen pressure range. This inspired us to investigate whether there exists a similar effect on hydrogen solubility and diffusivity for the substitution of Nb by Mo in Nb_40_Ti_30_Ni_30_ (all compositions in atomic percent). Nb_40_Ti_30_Ni_30_ is an attractive hydrogen permeable alloy in dual-phase Nb-Ti-Ni systems^26^. It exhibits strong HE resistance in a much larger hydrogen pressure range than that for Nb-based solid solution alloys. However, the hydrogen permeability of Nb_40_Ti_30_Ni_30_ is not quite satisfactory for the practical applications^48^. In this work, the substitution of Nb by Mo in Nb_40_Ti_30_Ni_30_ was investigated with respect to microstructural features and hydrogen dissolution, diffusion and permeation. It is expected that this compositional modification may improve the permeability and also enhance the HE resistance.

## Results and Discussion

### Microstructure

**Figure 1:**
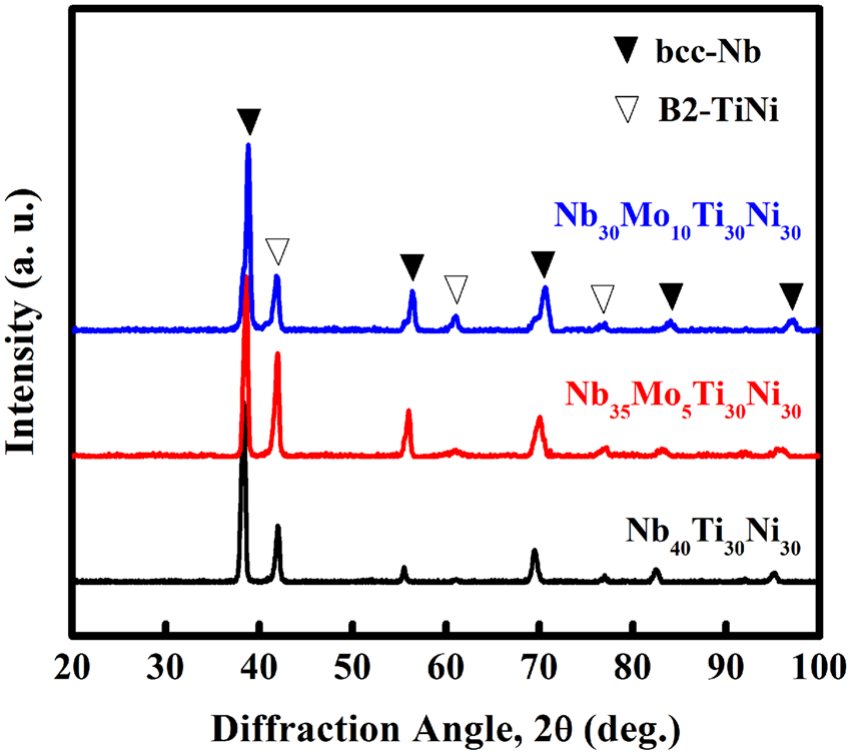
XRD patterns of as-cast Nb_40_Ti_30_Ni_30_, Nb_35_Mo_5_Ti_30_Ni_30_ and Nb_30_Mo_10_Ti_30_Ni_30_.

X-ray diffraction (XRD) patterns of as-cast Nb_40−x_Mo_x_Ti_30_Ni_30_ (x = 0, 5, 10) are shown in Fig. 1. All alloys consist of bcc-Nb solid solution and B2-TiNi, identified by the Bragg diffraction peaks. The substitution of Nb by Mo does not change the constituent phases. The locations of the B2-TiNi peaks are essentially identical, but the locations of the bcc-Nb peaks shift towards higher angles with increasing Mo content. Mo is mainly dissolved in Nb. The atomic radius of Mo (140.0 pm) is smaller than that of Nb (146.8 pm). According to Vegard‘s law the dissolution of Mo contracts the bcc-Nb crystal lattice, which results in the shift of the diffraction peak.

**Figure 2:**
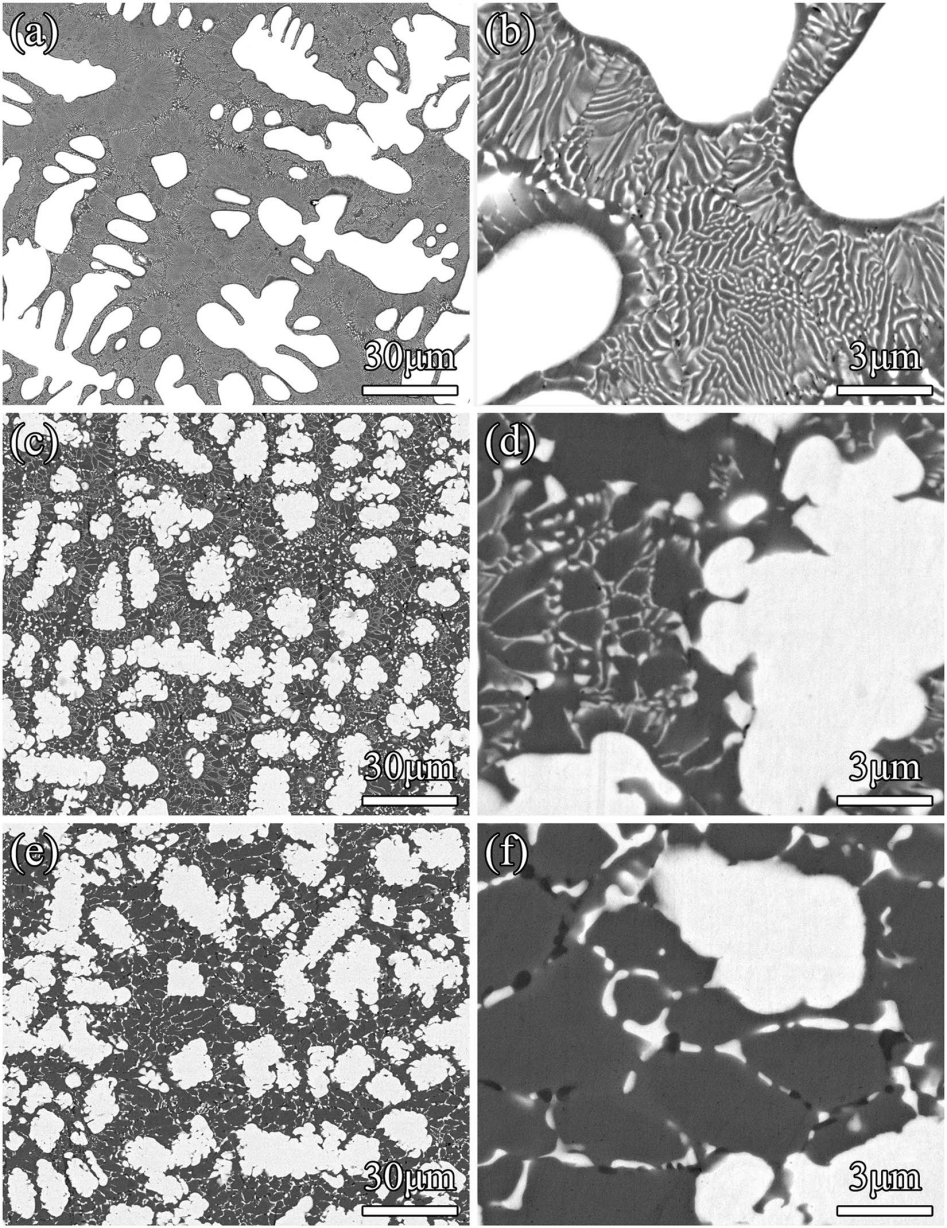
Back-scattered electron (BSE) micrographs of as-cast Nb_40_Ti_30_Ni_30_ (**a**), Nb_35_Mo_5_Ti_30_Ni_30_ (**c**) and Nb_30_Mo_10_Ti_30_Ni_30_ (**e**). (**b**,**d** and **f**) Show the magnified eutectic morphology in (**a**,**c** and **e**), respectively.

The microstructure of as-cast Nb_40−x_Mo_x_Ti_30_Ni_30_ (x = 0, 5, 10) is shown in Fig. 2. The white regions represent bcc-Nb and the dark regions represent B2-TiNi. Typical hypoeutectic microstructures, i.e. primary bcc-Nb dendrites surrounded by eutectic (bcc-Nb + B2-TiNi), are observed in all three as-cast alloys, as shown in Fig. 2(a,c and e). The substitution of Nb by Mo does not lead to a change in the microstructural sequence, but induces a change in growth morphology of bcc-Nb dendrites with less developed side branches. This may be attributed to the partial remelting of the primary bcc-Nb phase due to a retrograde solidus surface of the primary phase. In addition, the substitution of Nb by Mo results in a decrease in volume fraction of bcc-Nb in the eutectic, and a gradual increase in fraction of B2-TiNi, as seen in Fig. 2(b,d and f). Correspondingly, the eutectic structure changes from a typical lamellar (Fig. 2(b)) to a lamellar/degenerate (Fig. 2(e))) to a fully degenerate pattern (Fig. 2(f)).

### Hydrogen absorption

**Figure 3:**
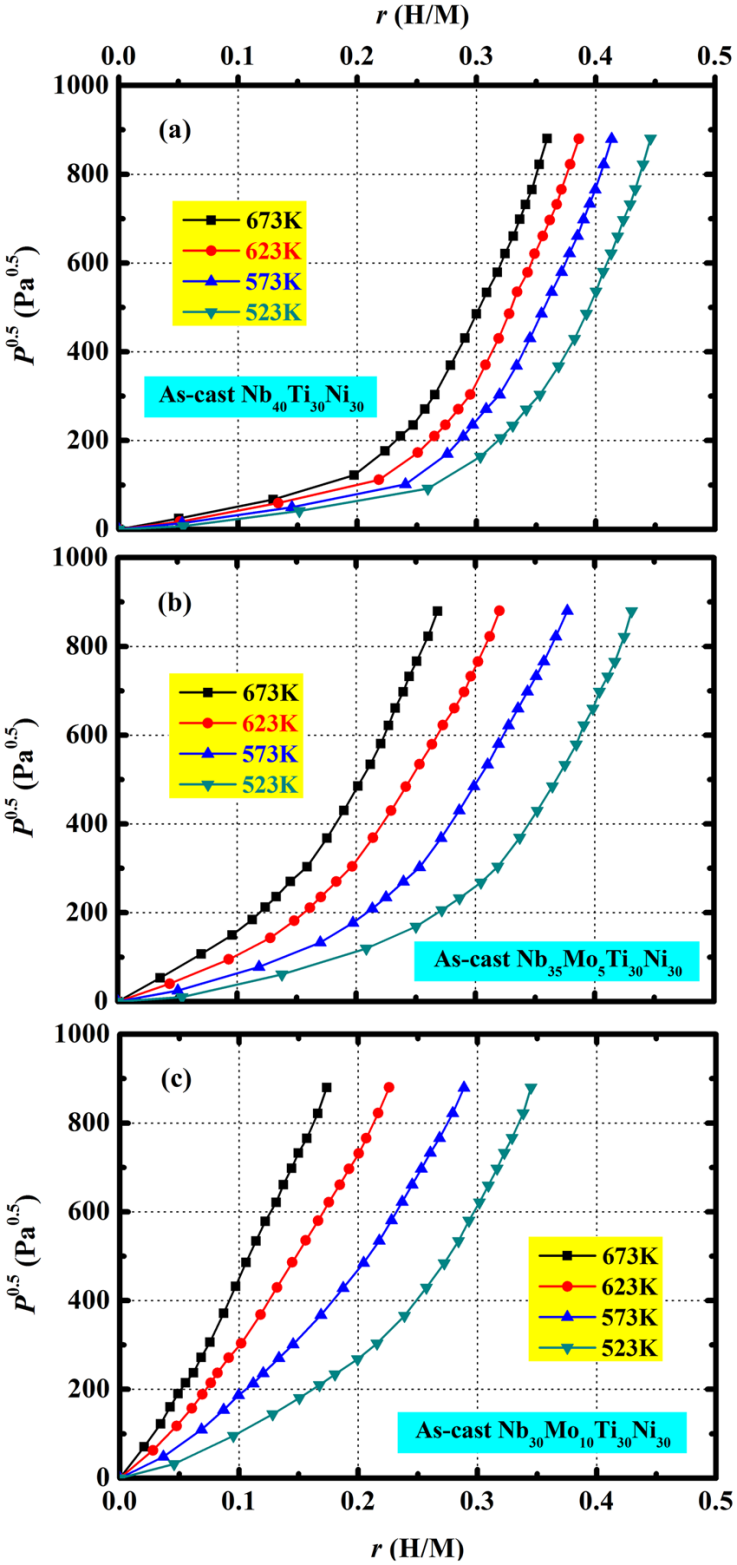
*PCT* curves for as-cast Nb_40_Ti_30_Ni_30_ (**a**), Nb_35_Mo_5_Ti_30_Ni_30_ (**b**) and Nb_30_Mo_10_Ti_30_Ni_30_ (**c**), measured at 523…673 K.

In Fig. 3, the equilibrium pressure-composition-temperature (*PCT*) curves for as-cast Nb_40−x_Mo_x_Ti_30_Ni_30_ (x = 0, 5, 10) are shown at the temperatures of 523…673 K. The hydrogen concentration *r* (H/M) is plotted against the square root of the hydrogen pressure *P* ^0.5^ in the form of a Sieverts’ plot. The hydrogen absorption behavior generally deviates from Sieverts’ law for all alloys except for that of Nb_30_Mo_10_Ti_30_Ni_30_ at 673 K. The hydrogen concentration in all alloys increases with increasing pressure and decreasing temperature. This reflects the exothermic behavior of hydrogen dissolution in (Nb, Mo)_40_Ti_30_Ni_30_. Hydrogen is mainly dissolved in the bcc-Nb of the dual-phase alloys, and Nb is one of the typical exothermic occluders^43^ whose hydrogen solubility clearly decreases with increasing temperature. Schmidt *et al.* ^49^ reported that TiNi also absorbs hydrogen exothermically, but the amount of absorbed hydrogen is negligible in the present pressure and temperature conditions.

From Fig. 3, it can also be seen that the substitution of Nb by Mo decreases the hydrogen solubility. This should be associated with the change in the elastic properties and the electronic structure induced by the substitutional Mo atoms in the host bcc-Nb lattice. The dissolution of Mo in Nb contracts the host bcc-Nb lattice (see Fig. 1), and a larger energy for elastic deformation is required for the absorption of H atoms. This corresponds to the interstitial site blocking mechanism^19^ that reduces hydrogen solubility (for an example in a V-Pd alloy, see ref. 19). The electronic structure is another important factor in establishing hydrogen solubility in metals. The electronegativity of Mo (2.16) is higher than that of Nb (1.6), but both values are lower than that of hydrogen (2.2). Hydrogen gains charge from each of the nearest neighbor metal atoms in the bcc-Nb lattice^50^. It can be expected that less charge is shared between the lattice and hydrogen when the neighboring Nb atoms are replaced by Mo. Correspondingly, the binding energy of hydrogen with the bcc-Nb lattice is reduced by the substitution of Nb by Mo. This inherently contributes to the reduction in hydrogen solubility.

### Hydrogen flux and permeability

**Figure 4:**
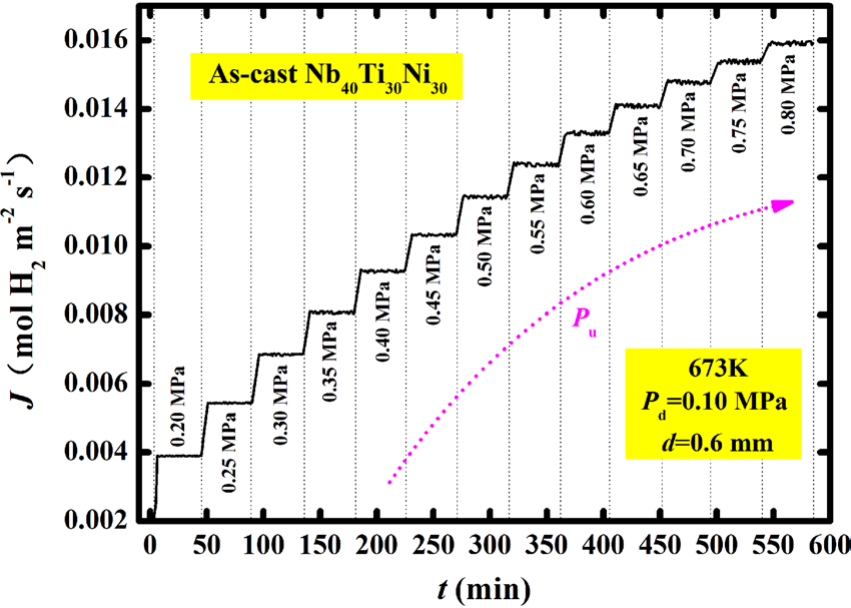
Measured hydrogen permeation flux (*J*’) as a function of time (*t*) at different upstream pressures (*P*_u_) at 673 K for the as-cast Nb_40_Ti_30_Ni_30_ alloy membrane.

In Fig. 4, the measured hydrogen permeation flux (*J*’) as a function of time (*t*) is shown for the as-cast Nb_40_Ti_30_Ni_30_ at 673 K as a representative case. The flux evidently increases with increasing the upstream pressure (*P*_u_). At each fixed *P*_u_, the flux is essentially stable after a short initial transient. This stable average flux is referred to as steady-state flux (*J*) under the given pressure and temperature conditions. Correspondingly, the relation between the *J* and *P*_u_ can be built, considering that the downstream hydrogen pressure (*P*_d_) is permanently maintained at 0.1 MPa.

**Figure 5:**
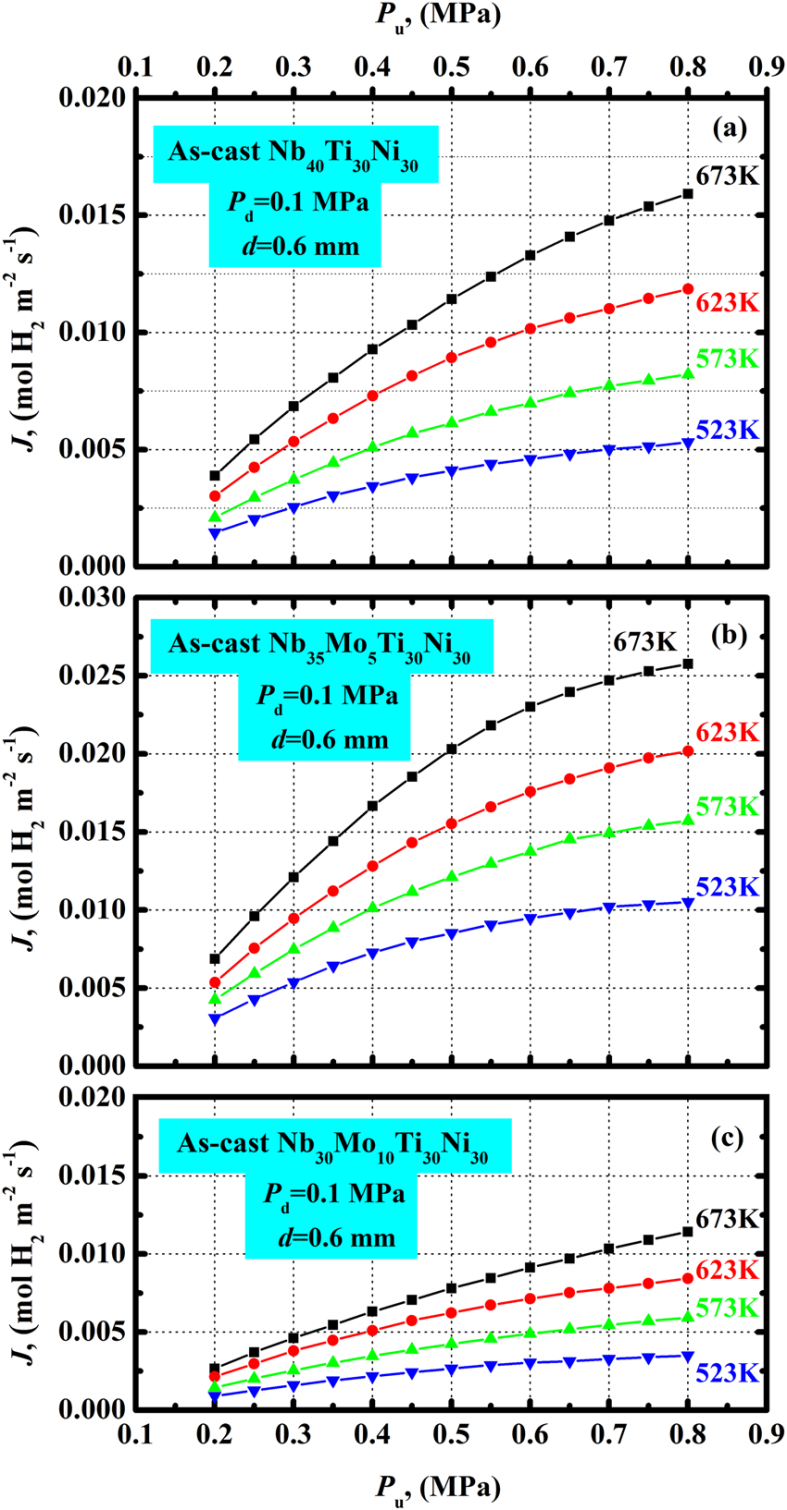
Steady-state hydrogen flux (*J*) plotted against the upstream hydrogen pressure (*P*_u_) for the as-cast Nb_40_Ti_30_Ni_30_ (**a**), Nb_35_Mo_5_Ti_30_Ni_30_ (**b**) and Nb_30_Mo_10_Ti_30_Ni_30_ (**c**) membranes, measured at 523…673 K.

In Fig. 5, the steady-state hydrogen permeation fluxes (*J*) at 523…673 K for membranes of as-cast Nb_40−x_Mo_x_Ti_30_Ni_30_ (x = 0, 5, 10) are shown. For each alloy membrane, the *J* values increase with increasing temperature or *P*_u_. At the same conditions, as-cast Nb_35_Mo_5_Ti_30_Ni_30_ exhibits the highest *J* values, whereas as-cast Nb_30_Mo_10_Ti_30_Ni_30_ exhibits the lowest values. The substitution of Nb by Mo increases the flux for a content of 5 at.% Mo, and distinctly reduces it for 10 at.% Mo. The membranes remain intact after the hydrogen permeation measurements at all temperatures and pressures, which were testified by the permeation test using Ar at room temperature. This indicates that all alloys are not susceptible to HE.

**Figure 6:**
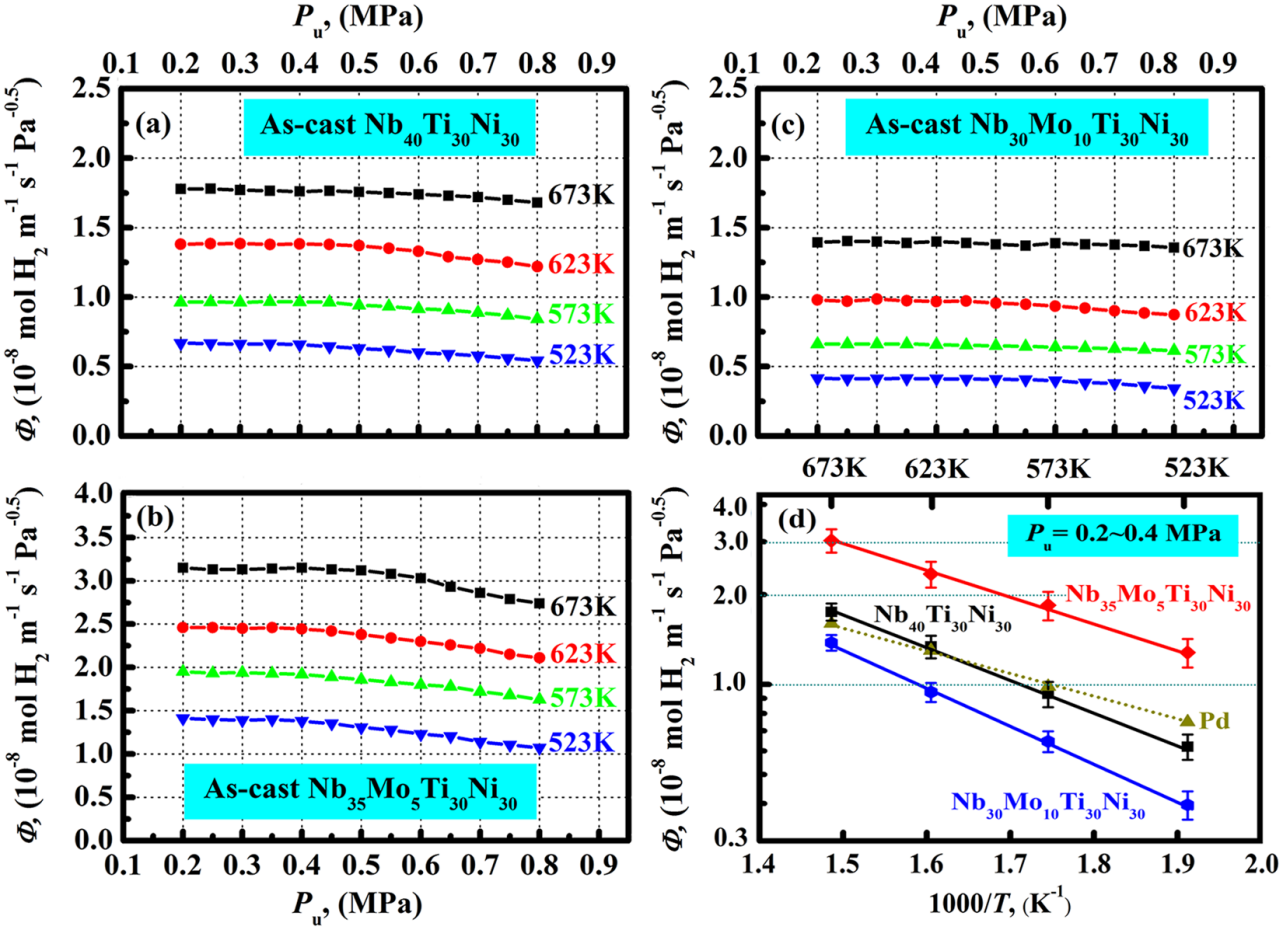
Calculated hydrogen permeability plotted against the upstream pressure (*P*_u_) for the as-cast Nb_40_Ti_30_Ni_30_ (**a**), Nb_35_Mo_5_Ti_30_Ni_30_ (**b**) and Nb_30_Mo_10_Ti_30_Ni_30_ (**c**) alloy membranes at 523…673 K, and temperature dependence of *Φ* in the form of an Arrhenius plot in the pressure range of 0.2…0.4 MPa (**d**).

In earlier work on Nb-based dual-phase alloy membranes^26–36^, the hydrogen flux (*J*) was generally transformed to permeability (*Φ*) (in mol H_2_ m^−1^s^−1^Pa^−0.5^) using the relationship *Φ* = *J*·*d*/Δ*P* ^0.5^. In Fig. 6(a–c), the calculated hydrogen permeabilities of the as-cast Nb_40−x_Mo_x_Ti_30_Ni_30_ (x = 0, 5, 10) alloys are plotted against the upstream hydrogen pressure. The *Φ* values of each membrane evidently increase with increasing temperature. At a given temperature, *Φ* is essentially constant at a *P*_u_ of 0.2…0.4 MPa for all membranes, but gradually exhibits pressure dependence at higher *P*_u_ values (>0.4 MPa), decreasing with increasing *P*_u_. The constant *Φ* value is attributed to the linear relation between *C* and *P* ^0.5^ (i.e. *C* = *K* · *P* ^0.5^ + *α*) and the linear relation between *J*·*d* and Δ*P* ^0.5^ in the pressure range of 0.1…0.4 MPa at a given temperature. This is commonly found in Nb-based dual-phase alloy membranes^27, 31, 34–36^. The temperature dependence of *Φ* in the Arrhenius plot follows a straight line for each membrane in the low hydrogen pressure range, as shown in Fig. 6(d). The *Φ* values of as-cast Nb_40_Ti_30_Ni_30_ are slightly higher than those of pure Pd at high temperatures. The substitution of Nb by Mo increases the *Φ* values for 5 at.% Mo, but distinctly reduces the values for 10 at.% Mo. The as-cast Nb_35_Mo_5_Ti_30_Ni_30_ exhibits an attractive hydrogen permeability, particularly 3.13 × 10^−8^ mol H_2_ m^−1^s^−1^Pa^−0.5^ at 673 K. This is ~1.96 times that of pure Pd. At higher *P*_u_ values (>0.4 MPa), the decrease of *Φ* with increasing *P*_u_ indicates a deviation from ideal Sieverts’-type behavior, which is expected from the hydrogen absorption data.

### Hydrogen diffusion

The above results clearly show that the hydrogen permeability of (Nb, Mo)_40_Ti_30_Ni_30_ alloys is sensitive to the Mo content. This suggests that the substitution of Nb by Mo also induces different hydrogen diffusion behavior in alloys. The apparent hydrogen diffusion coefficient can be calculated by *D* = *J*·*d*/Δ*C* according to Fick’s first law. Here, Δ*C* is the difference of hydrogen concentration between the upstream and downstream surfaces of the membrane. *D* is the concentration dependent diffusion coefficient that can be further associated with the concentration independent diffusion coefficient (D*):

1 $$D={D}^{\ast }{(\frac{\partial \mathrm{ln}{p}^{1/2}}{\partial \mathrm{ln}r})}_{T}={D}^{\ast }f(r)$$

Here, *f*(*r*) is the thermodynamic factor that can be calculated by the hydrogen absorption data in Fig. 3, as described previously^36^.

The concentration dependent apparent diffusion coefficient (*D*) takes into account the chemical potential gradient of hydrogen as the thermodynamic driving force for diffusion, which reveals the average/overall diffusion behavior of hydrogen in membranes. The concentration independent hydrogen diffusion coefficient (D*) is known as Einstein’s diffusion coefficient or intrinsic diffusion coefficient. D* is strictly only applicable in infinitely dilute systems with a random component in the movement of hydrogen atoms, which accounts for the interaction of hydrogen and the metal lattice. Dolan *et al.* ^44^ first put forward a method of calculating concentration dependent and concentration independent hydrogen diffusion coefficients of V-Ni alloy membranes by correlating hydrogen absorption and flux data. This method was extended to evaluate hydrogen diffusion behavior of several other V-based hydrogen permeable alloys^51^ and also of the present (Nb, Mo)_40_Ti_30_Ni_30_ alloys.

**Figure 7:**
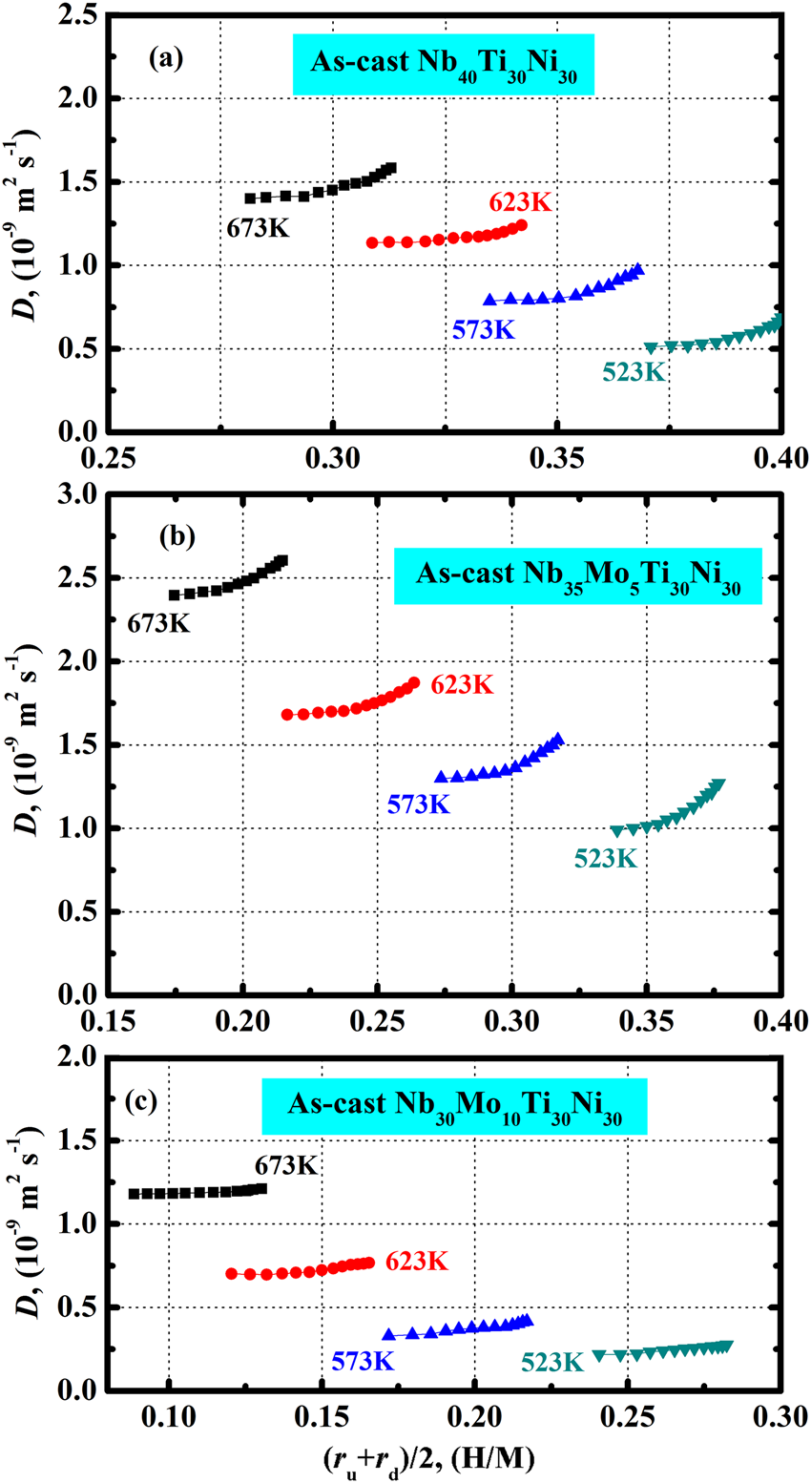
Concentration dependent apparent hydrogen diffusion coefficient (*D*) for the as-cast Nb_40_Ti_30_Ni_30_ (**a**), Nb_35_Mo_5_Ti_30_Ni_30_ (**b**) and Nb_30_Mo_10_Ti_30_Ni_30_ (**c**) alloy membranes at 523…673 K. The hydrogen concentration is given by (*r*_u_ + *r*_d_)/2 which corresponds to an average H/M values at the upstream and downstream surfaces of each membrane.

In Fig. 7, the concentration dependent *D* for as-cast Nb_40−x_Mo_x_Ti_30_Ni_30_ (x = 0, 5, 10) is shown. The *D* values for all membranes increase with increasing temperature. At each temperature, the *D* values obviously increase with increasing hydrogen concentration (*r*). This originates from the change in the electronic structure of bcc-Nb with the gradual addition of H. It was found that the *d* bands of Nb were modified by the interaction with H, resulting in the appearance of H-induced new energy states^52^. This potentially reduces the activation energy for interstitial hopping of H in the bcc-Nb lattice. In addition, the mobility of hydrogen in the metal lattice is sensitive to the hydrogen concentration in terms of the self-trapping mechanism^53^. At low hydrogen concentrations, the relaxation of the lattice tends to be complete, and hydrogen mainly resides at deep potential sites. As the hydrogen concentration increases, lattice relaxation turns to be less advanced, implying that the average hydrogen potential becomes less deep, so that hydrogen can overcome the potential barrier more easily. Thus, the mobility of hydrogen increases with hydrogen concentration. The reduction in the diffusion activation energy and the increase in mobility altogether contribute to the gradual increase of *D* with *r*.

**Figure 8:**
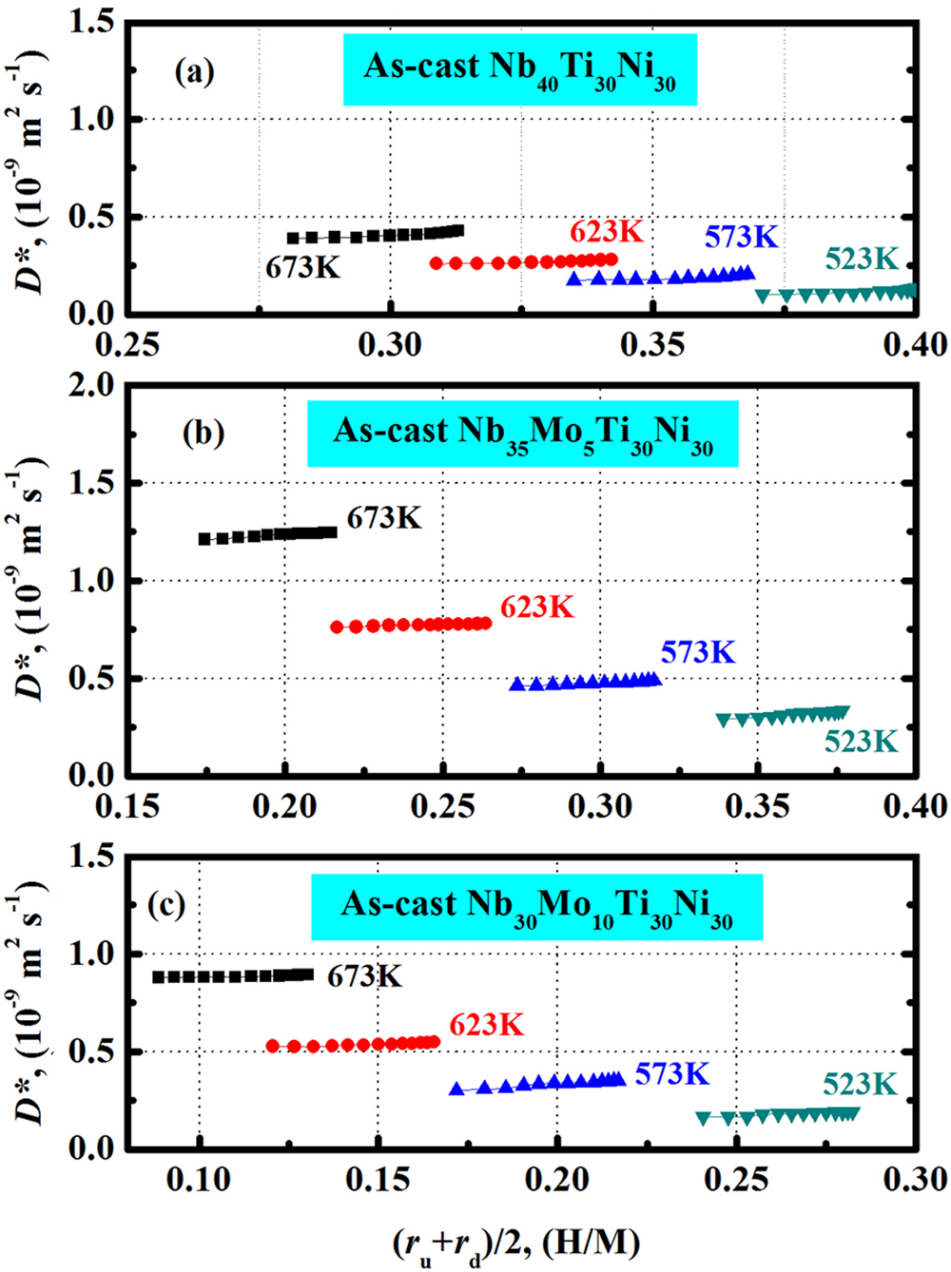
Concentration independent hydrogen diffusion coefficient (D*) for the as-cast Nb_40_Ti_30_Ni_30_ (**a**), Nb_35_Mo_5_Ti_30_Ni_30_ (**b**) and Nb_30_Mo_10_Ti_30_Ni_30_ (**c**) alloy membranes at 523…673 K.

**Figure 9:**
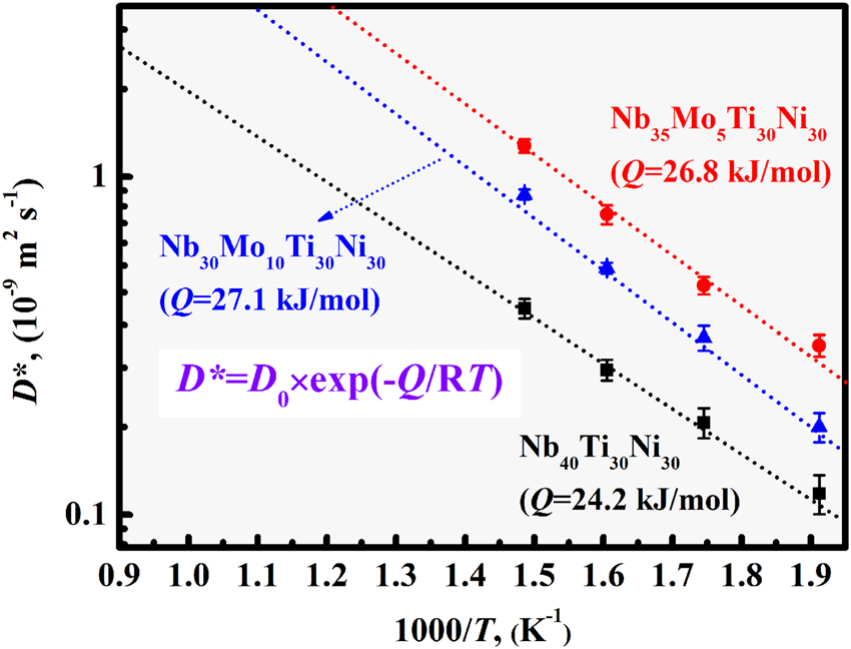
Temperature dependence of the average D*, shown in the form of an Arrhenius plot for as-cast Nb_40−x_Mo_x_Ti_30_Ni_30_ (x = 0, 5, 10) at 523…673 K. The activation energy (*Q*) for hydrogen diffusion and the pre-exponential factor (*D*_0_) in each case were determined by linear regression.

From Fig. 7, it can also be seen that the substitution of Nb by Mo increases the apparent hydrogen diffusion coefficient (*D*) for 5 at.% Mo, but reduces the values for 10 at.% Mo. This should be associated with the responses of D* and *f*(*r*) to the substitution according to equation (1). Fig. 8 shows the concentration independent D* for the three alloy membranes at 523…673 K. The D* values are almost constant at a fixed temperature for each membrane, but there is a slight trend toward higher D* values with increasing *r*. The average D* in each case follows a linear temperature dependence in the Arrhenius plot, as shown in Fig. 9. The activation energy (*Q*) of hydrogen diffusion for a dilute hydrogen concentration, and the diffusion pre-exponential factor (*D*_0_) can be determined. From Fig. 9 it is concluded that the addition of Mo in Nb increases the average D*, in a more pronounced manner for x = 5 than for x = 10. This particular change mainly originates from the difference in the *D*_0_ values, since there is no significant difference in the *Q* values for the three alloys. *D*_0_ can be expressed as ${D}_{0}=\frac{{\lambda }^{2}}{6}Z\nu \cdot \exp (\frac{{S}_{B}+{S}_{W}}{k})$, where λ, Z, *v*, *S*_*B*_, *S*_*W*_ and *k* are jump width, correlation number, jump frequency, entropy of vacancy formation, entropy of vacancy migration and Boltzmann’s constant, respectively. The substitution of Nb by Mo must induce diverse effects on the parameters in the expression of *D*_0_, such that the *D*_0_ and also D* values are sensitive to the Mo content. Overall, the addition of Mo in Nb increases the intrinsic hydrogen diffusion coefficient (D*), which provides a positive contribution to the apparent hydrogen diffusion coefficient (*D*). However, the hydrogen solubility in (Nb, Mo)_40_Ti_30_Ni_30_ alloys is reduced by the substitution of Nb by Mo (see Fig. 3). This results in the reduction of the thermodynamic factor *f*(*r*) that acts as driving force for hydrogen diffusion. Consequently, the continuous decrease in *f*(*r*) and the particular change in the D* altogether induce the diverse trend of *D* with the addition of Mo.

### Hydrogen embrittlement (HE)

**Figure 10:**
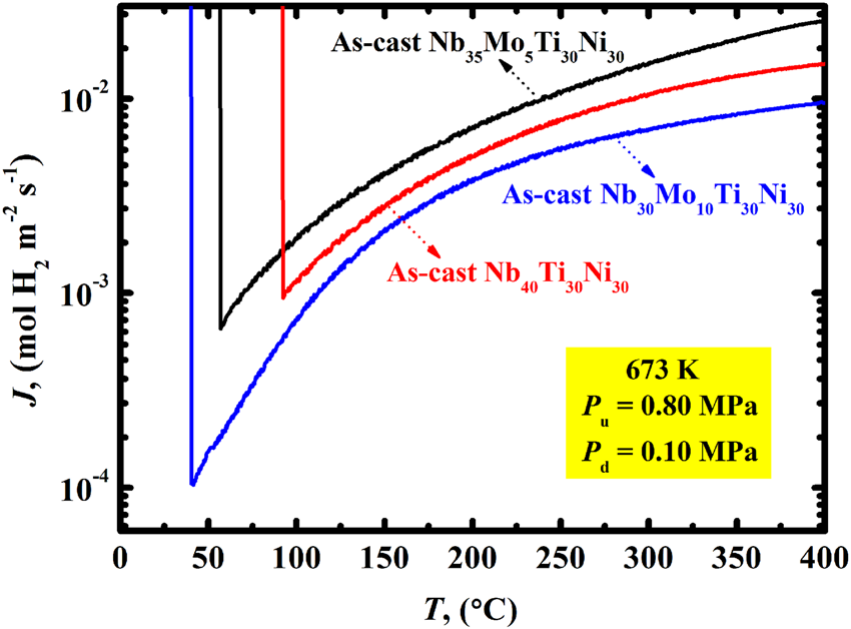
Measured hydrogen permeation flux during cooling from 400 °C at a cooling rate of 2 °C min^−1^ for the as-cast Nb_40_Ti_30_Ni_30_, Nb_35_Mo_5_Ti_30_Ni_30_ and Nb_30_Mo_10_Ti_30_Ni_30_ alloy membranes. Before cooling, the alloys were placed in a hydrogen permeation atmosphere of 0.7 MPa pressure difference at 400 °C for 4 h.

The hydrogen permeation tests in Figs 4 and 5 indicate that as-cast Nb_40−x_Mo_x_Ti_30_Ni_30_ (x = 0, 5, 10) alloy membranes exhibit an appropriate tolerance to HE at 523…673 K. A further evaluation of HE sensitivity was conducted by hydrogen permeation under controlled cooling conditions. Such evaluations have technical relevance, particularly concerning membranes during sustained operation with intermittent thermal cycling. Figure 10 shows the measured hydrogen flux as a function of temperature for the membranes. The flux decreases with decreasing temperature in all cases. An abrupt increase in flux is observed for Nb_40_Ti_30_Ni_30_, Nb_35_Mo_5_Ti_30_Ni_30_ and Nb_30_Mo_10_Ti_30_Ni_30_ at ~96 °C, ~55 °C, ~37 °C, respectively. This originates from crack formation due to HE failure. The fracture temperature gradually decreases with increasing the Mo content. This indicates that the substitution of Nb by Mo improves the HE resistance, which can straightforwardly be attributed to the decrasing hydrogen solubility (see Fig. 3).

It can be expected that the three alloy membranes investigated in the present study are robust under the operating conditions representative for industrial applications such as water gas shift reaction (typically at 0.7…1 MPa and 300…500 °C). However, hydrogen should be evacuated from the membrane at temperatures above the critical fracture temperatures mentioned above. Although the substitution of Nb by Mo cannot enable the membranes to be cycled to room temperature under H_2_ atmosphere, it distinctly lowers the temperature to which they can be cycled. Overall, Nb_35_Mo_5_Ti_30_Ni_30_ exhibits the best balance between hydrogen permeability and HE resistance. This alloy thus has high potential to serve as non-Pd alloy membrane for hydrogen separation.

## Conclusions

Nb_40_Ti_30_Ni_30_ is one of the competitive alternatives to Pd-based alloy membranes for hydrogen separation and purification, while further enhancement of hydrogen permeability and embrittlement resistance is necessary to ensure its practical application. A promising strategy is compositional modification with the aim of controlling the hydrogen solubility to a point where embrittlement is inhibited, while the hydrogen diffusivity is increased. The present work shows that the substitution of Nb by Mo in Nb_40_Ti_30_Ni_30_ does not lead to a change in the microstructural evolution sequence, while obviously hydrogen solubility is reduced. The apparent hydrogen diffusivity and permeability increase with small Mo additions, but decease with larger ones. This can be attributed to the sensitive response of intrinsic hydrogen diffusivity and thermodynamic factor to the Mo content. Overall, Nb_35_Mo_5_Ti_30_Ni_30_ exhibits a combined enhancement of hydrogen permeability and embrittlement resistance as compared to Nb_40_Ti_30_Ni_30_. Mo is a desirable alloying element in Nb for an excellent balance of permeability and embrittlement resistance.

## Experimental

### Samples

About 40 g ingots of Nb_40−x_Mo_x_Ti_30_Ni_30_ (x = 0, 5, 10) alloys were prepared by an arc melting method in purified Ar atmosphere using Nb, Mo, Ti and Ni (99.99 mass% purity for all) as raw materials. All ingots were melted 6 times for compositional homogeneity. Phase identification of the as-cast samples was conducted by X-ray diffraction (XRD). Microstructural observations were carried out using scanning electron microscopy (SEM) in the back-scattered electron (BSE) mode.

### Hydrogen absorption and permeation

Disk samples of 12 mm in diameter were cut from the as-cast ingots using a spark erosion wire cutting machine. Both surfaces of the samples were ground with SiC paper (400–2000 grid) and then polished using 0.5 μm Al_2_O_3_ powder. The final thickness (*d*) of the samples was 0.6 mm. A pure Pd layer of 190 nm in thickness was then coated on both sides of the sample by a magnetron sputtering device for avoiding oxidation and enhancing the dissociation/recombination of hydrogen molecules during the hydrogen absorption and permeation tests.

Hydrogen absorption of as-cast Nb_40−x_Mo_x_Ti_30_Ni_30_ (x = 0, 5, 10) was characterized using a Sieverts-type apparatus. The equilibrium pressure-composition-temperature (*PCT*) data were measured at pressures in the range of 0.01–1.0 MPa at 523 K, 573 K, 623 K and 673 K, respectively. More details about the experimental procedure can be found in refs 17 and 18.

Samples for the hydrogen permeation tests were sandwiched by two copper gaskets and fixed in a gas permeation device. Both sides of the sample were evacuated, heated to 673 K in a vacuum and kept for 20 minutes. Hydrogen gas (99.99999 mass% purity) was introduced at pressures of 0.2–0.8 MPa and 0.1 MPa to the upstream and downstream sides of the sample, respectively. The hydrogen permeation flux through the sample was measured using a mass flow meter. The measurements were repeated at 623 K, 573 K and 523 K. In order to investigate the sensitivity to HE for the as-cast alloys under H_2_ atmosphere, the hydrogen permeation tests were conducted under controlled cooling conditions. The samples were first subjected to hydrogen permeation at a hydrogen pressure difference of 0.7 MPa at 400 °C for 4 h, followed by cooling to room temperature at a cooling rate of 2 °C min^−1^. The variation of the hydrogen permeation flux with time and temperature was measured. An abrupt increase in the hydrogen flux indicates the formation of cracks in the sample due to HE^16^.

## References

[1] Mordkovich VZ, Baichtock YK, Sosna MH (1992). The large scale production of hydrogen from gas mixtures. Platinum Met. Rev..

[2] Hara S, Haraya K, Barbieri G, Drioli E (2008). Reaction rate profiles in long palladium membrane reactors for methane steam reforming. Desalination.

[3] Hara S, Barbieri G, Drioli E (2006). Limit conversion of a palladium membrane reactor using counter-current sweep gas on methane steam reforming. Desalination.

[4] Gallucci F, Tosti S, Basile A (2008). Pd-Ag tubular membrane reactors for methane dry reforming: a reactive method for CO_2_ consumption and H_2_ production. J. Membr. Sci..

[5] Borgognoni F, Tosti S, Vadrucci M, Santucci A (2013). Combined methane and ethanol reforming for pure hydrogen production through Pd-based membranes. Int. J. Hydrog. Energy.

[6] Dolan MD, Donelson R, Dave NC (2010). Performance and economics of a Pd-based planar WGS membrane reactor for coal gasification. Int. J. Hydrog. Energy.

[7] Iyoha O (2007). Wall-catalyzed water-gas shift reaction in multi-tubular Pd and 80 wt%Pd-20 wt%Cu membrane reactors at 1173 K. J. Membr. Sci..

[8] Pinacci P, Brogli M, Vallia C, Capannelli G, Comite A (2010). Evaluation of the water gas shift reaction in a palladium membrane reactor. Catal. Today.

[9] Shirasaki Y (2009). Development of membrane reformer system for highly efficient hydrogen production from natural gas. Int. J. Hydrog. Energy.

[10] Nozaki T, Hatano Y, Yamakawa E, Hachikawa A, Ichinose K (2010). Improvement of high temperature stability of Pd coating on Ta by HfN intermediate layer. Int. J. Hydrog. Energy.

[11] Tosti S (2015). Production of hydrogen in a Pd-membrane reactor via catalytic reforming of olive mill wastewater. Chem. Eng. J..

[12] Tosti S, Fabbricino M, Pontoni L, Palma V, Ruocco C (2016). Catalytic reforming of olive mill wastewater and methane in a Pd-membrane reactor. Int. J. Hydrog. Energy.

[13] Hatlevik O (2010). Palladium and palladium alloy membranes for hydrogen separation and production: history, fabrication strategies, and current performance. Sep. Purif. Technol..

[14] Nishimura C, Komaki M, Hwang S, Amano M (2002). V-Ni alloy membranes for hydrogen purification. J. Alloys Compd..

[15] Paglieri SN (2008). Development of membranes for hydrogen separation: Pd coated V-10Pd. Energy Mater.: Mater. Sci. Eng. Energy Syst..

[16] Dolan MD (2011). Hydrogen transport through V_85_Ni_10_M_5_ alloy membranes. J. Membr. Sci..

[17] Dolan MD, McLennan KG, Song G, Liang D, Kellam ME (2013). The effect of Ti on hydrogen absorption and diffusivity in V-Ti-Al alloy membranes. J. Membr. Sci..

[18] Kim KH, Park HC, Lee J, Cho E, Lee SM (2013). Vanadium alloy membranes for high hydrogen permeability and suppressed hydrogen embrittlement. Scrip. Mater..

[19] Alimov VN, Busnyuk AO, Notkin ME, Peredistov EY, Livshits AI (2014). Substitutional V-Pd alloys for the membranes permeable to hydrogen: Hydrogen solubility at 150–400 °C. Int. J. Hydrog. Energy.

[20] Alimov VN, Busnyuk AO, Notkin ME, Peredistov EY, Livshits AI (2015). Hydrogen transport through V-Pd alloy membranes: Hydrogen solution, permeation and diffusion. J. Membr. Sci..

[21] Komiya K, Shinzato Y, Yukawa H, Morinaga M, Yasuda I (2005). Measurement of hydrogen permeability of pure Nb and its alloys by electrochemical method. J. Alloys Compd..

[22] Watanabe N (2009). Alloying effects of Ru and W on the resistance to hydrogen embrittlement and hydrogen permeability of Nb. J. Alloys Compd..

[23] Watanabe N (2009). Mechanical properties in hydrogen atmosphere and hydrogen permeability of Nb-W-Ta alloys for hydrogen permeable membrane. J. Jpn. Inst. Met..

[24] Awakura Y, Nambu T, Matsumoto Y, Yukawa H (2011). Hydrogen solubility and permeability of Nb-W-Mo alloy membrane. J. Alloys Compd..

[25] Rothenberger KS (2003). Evaluation of tantalum-based materials for hydrogen separation at elevated temperatures and pressures. J. Membr. Sci..

[26] Hashi K, Ishikawa K, Matsuda T, Aoki K (2004). Hydrogen permeation characteristics of multi-phase Ni-Ti-Nb alloys. J. Alloys Compd..

[27] Wang W, Ishikawa K, Aoki K (2010). Microstructural change-induced lowering of hydrogen permeability in eutectic Nb-TiNi alloy. J. Membr. Sci..

[28] Magnone E, Jeona SI, Park JH, Fleury E (2011). Relationship between microstructure and hydrogen permeation properties in the multiphase Ni_21_Ti_23_Nb_56_ alloy membranes. J. Membr. Sci.

[29] Yan EH (2014). Design of hydrogen permeable Nb-Ni-Ti alloys by correlating the microstructures, solidification paths and hydrogen permeability. Int. J. Hydrog. Energy.

[30] Saeki Y, Yamada Y, Ishikawa K (2014). Relationship between hydrogen permeation and microstructure in Nb-TiNi two-phase alloys. Int. J. Hydrog. Energy.

[31] Li XZ (2015). Changes in microstructure, ductility and hydrogen permeability of Nb-(Ti, Hf)Ni alloy membranes by the substitution of Ti by Hf. J. Membr. Sci..

[32] Hashi K, Ishikawa K, Matsuda T, Aoki K (2006). Microstructure and hydrogen permeability in Nb-Ti-Co multiphase alloys. J. Alloys Compd..

[33] Luo W, Ishikawa K, Aoki K (2012). Highly hydrogen permeable Nb-Ti-Co hypereutectic alloys containing much primary bcc-(Nb, Ti) phase. Int. J. Hydrog. Energy.

[34] Li XZ (2015). Substantial enhancement of hydrogen permeability and embrittlement resistance of Nb_30_Ti_25_Hf_10_Co_35_ eutectic alloy membranes by directional solidification. J. Membr. Sci..

[35] Li XZ (2016). Microstructure dependent hydrogen permeability in eutectic Nb_30_Ti_35_Co_35_. Int. J. Hydrog. Energy.

[36] Li XZ (2016). Hydrogen transportation behavior of as-cast, cold rolled and annealed Nb_40_Ti_30_Co_30_ alloy membranes. J. Membr. Sci..

[37] Adams TM, Mickalonis J (2007). Hydrogen permeability of multiphase V-Ti-Ni metallic membranes. Mater. Lett..

[38] Song G, Dolan MD, Kellam ME, Liang D, Zambelli S (2011). V-Ni-Ti multi-phase alloy membranes for hydrogen purification. J. Alloys Compd..

[39] Jeon SI, Magnone E, Park JH, Lee Y (2011). The effect of temperature and pressure on the hydrogen permeation through Pd-coated Ti_26_Ni_21_V_53_ alloy membrane under different atmospheres. Mater. Lett..

[40] Hashi K, Ishikawa K, Matsuda T, Aoki A (2005). Hydrogen permeation characteristics of (V, Ta)-Ti-Ni alloys. J. Alloys Compd..

[41] Luo W, Ishikawa K, Aoki K (2008). Hydrogen permeable Ta-Ti-Ni duplex phase alloys with high resistance to hydrogen embrittlement. J. Alloys Compd..

[42] Miller, C. L., Cicero, D. C. & Ackiewicz, M. Hydrogen from Coal Program: Research, Development and Demonstration Plan for the Period 2007 through 2016, United States Department of Energy, National Energy Technology Laboratory (2007).

[43] Dolan MD (2010). Non-Pd bcc alloy membranes for industrial hydrogen separation. J. Membr. Sci..

[44] Dolan MD, McLennan KG, Way JD (2011). Diffusion of Atomic Hydrogen through V-Ni Alloy Membranes under Nondilute Conditions. J. Phys. Chem. C.

[45] Dolan MD, Song G, McLennan KG, Kellam ME, Liang D (2012). The effect of Ti on the microstructure, hydrogen absorption and diffusivity of V-Ni alloy membranes. J. Membr. Sci..

[46] Awakura Y, Nambu T, Matsumoto Y, Yukawa H (2011). Hydrogen solubility and permeability of Nb-W-Mo alloy membrane. J. Alloys Compd..

[47] Tsuchimoto K, Yukawa H, Nambu T, Matsumoto Y, Murata Y (2013). Design of Nb-W-Mo alloy membrane for hydrogen separation and purification. J. Alloys Compd..

[48] Tang HX, Ishikawa K, Aoki K (2008). Microstructure, ductility and hydrogen permeability of Nb-Ti-Zr-Ni Alloys. Mater. Trans..

[49] Schmidt R, Schlereth M, Wipf H, Assmus W, Müllner M (1989). Hydrogen solubility and diffusion in the shape-memory alloy NiTi. J. Phys. Condens. Mater..

[50] Aboud S, Wilcox J (2010). A Density Functional Theory Study of the Charge State of Hydrogen in Metal Hydrides. J. Phys. Chem. C.

[51] Dolan MD, Kellam ME, McLennan KG, Liang D, Song G (2013). Hydrogen transport properties of several vanadium-based binary alloys. *Int*. *J*. Hydrog. Energy.

[52] Peterman D, Misemer D, Weaver J, Peterson D (1983). Electronic structure of metal hydrides, VI. Photoemission studies and band theory of VH, NbH, and TaH. Phys. Rev. B.

[53] Hara S, Shimano E, Tsuji T (2012). Hydrogen diffusion coefficient and mobility in palladium as a function of equilibrium pressure evaluated by permeation measurement. J. Membr. Sci..

